# Exclusion zone and heterogeneous water structure at ambient temperature

**DOI:** 10.1371/journal.pone.0195057

**Published:** 2018-04-18

**Authors:** Seong G. Hwang, Jun Ki Hong, Abha Sharma, Gerald H. Pollack, GunWoong Bahng

**Affiliations:** 1 Department of Animal Life and Environmental Science, Hankyong National University, Anseong, Korea; 2 Center of Excellence in Wireless and Information Technology, Korea, Incheon, Korea; 3 Department of Bioengineering, University of Washington, Seattle, United States of America; 4 Department of Mechanical Engineering, The State University of New York, Korea, Incheon, Korea; University of California, Merced, UNITED STATES

## Abstract

Earlier studies have reported the formation of an exclusion zone devoid of microspheres at the interface of water with a hydrophilic surface such as Nafion^®^ or the hydrophilic ceramic powder. We now report the formation of a ‘three-dimensional cell-like structured exclusion zone’ in water prepared by two different methods. In the first, the hydrophilic powder was agitated with deionized water and allowed to rest (contact method). Subsequently, the ‘powder-supernatant water’ was collected and termed ‘contact water’. In the second method, deionized water in a closed container was kept in the close vicinity of the hydrophilic powder for an extended time-period and it was termed ‘non-contact water’. The two kinds of waters were tested by standard methods for various physical properties. In addition, we carried out cryogenic scanning-electron microscopy of frozen samples of the two kinds of water. The powder-supernatant water showed a cell-like heterogeneous ice structure with the high-density exclusion-zone water forming the walls of a cell-like structure. A similar cell-like ice structure was formed for water treated with the hydrophilic powder in a non-contact manner; the unit cell size depended on the ‘degree of structure’ in the water. When highly structured, the unit cell size was smaller with a concurrently enhanced dielectric constant and reduced redox potential. It was found that the electrical properties are more sensitive to the change in water structure compared to other physical properties such as surface tension, density, and specific heat. Based on our findings of an electric potential difference between the heterogeneous structured water and the ordinary water, we propose a new model to explain the relationship between heterogeneous, structured water and its electrical properties.

## 1. Introduction

Water, so essential to life, is assumed to be homogeneous with a continuous, uniform, and symmetrical tetrahedral-like-structure at ambient temperatures [1, 2]. Recently, however, high-resolution X-ray emission spectroscopy and other methods have suggested a heterogeneous model for water, composed of high-density water and low-density water [3–9]. In this heterogeneous model, high-density water is assumed to be in strongly hydrogen-bonded chains or rings embedded in a disordered cluster network connected by weak hydrogen bonds that forms the low-density water. However, the reality and detailed overall heterogeneous structure of water is not clear until this time.

The discovery of large, solute-free interfacial zones, aptly termed as “exclusion zones” (EZs) next to hydrophilic surfaces in aqueous solutions [10] as well as several polar liquids [11] indicate regions of water that are more ordered and denser than bulk water [12]. This may provide a clue to the understanding of the origin of high-density water. Here, we propose a hypothesis that the high-density water in heterogeneous structure and the exclusion zone are the same ones. To explore the possibility of this hypothesis, we have utilized cryogenic scanning electron microscopy to estimate the three-dimensional heterogeneous structure of water.

While Nafion^®^ remains an extensively used nucleating surface in prior studies [10–12], recently we explored the formation of EZs around another highly hydrophilic ceramic powder, QELBY^®^ [13]. Manufactured from the feldspar family of clay minerals through a special process, it’s composition and details are presented in a Korean patent (KP 10–1172018) [14]. When it was mixed with water, it was observed under an optical microscope that the microspheres were expelled out from the surface of the hydrophilic particles which indicate the formation of EZ. From this fact, it is anticipated that the EZs can be developed into a three-dimensional shape to form a heterogeneous structure of bulk water. The current study evaluates the results of cryogenic scanning-electron microscopic studies on a possible three-dimensional cell-like structured exclusion zone formed around the hydrophilic particles.

One of the most striking features of EZs is the generation of electric potential as high as ~200 mV between the EZ and the outside of it [15]. Also, it was observed that the proton concentration increases outside of the boundary between the EZ and ordinary water. Therefore, it is expected that the electric properties of heterogeneous water composed of EZ and ordinary water would be changed more sensitively compared to many other properties as the water is structured. In this study, redox potential and dielectric constant were evaluated with the formation of structured water.

We also examined the development of an electric potential difference across electrodes placed in structured water and ordinary water, respectively. A specially formulated container with built-in separators of different materials was used to observe a change in the polarity depending on the separator materials, Nafion^®^ or aluminum foil. Since Nafion^®^ is a good proton conductor and aluminum is a good electric conductor, reversed polarity indicates the formation of heterogeneous water structure composed of EZ and ordinary water.

The energy source for the formation of EZ is known to be the light including infrared waves. Like other ceramic materials, the hydrophilic powder, QELBY^®^, emits a high-level of near- and far-infrared (FIR) radiation that is absorbed by water and can influence water structure and properties [16–20]. We have reported these and other findings on the energizing effect of the hydrophilic powder in both the ‘contact’ and ‘non-contact’ seed model of germination and early sapling growth [13]. The similar effect was also observed in bioactivity of cells [21]. To evaluate the possibility that the non-contact biological effect of the infrared wave is due to the formation of EZs, the structure of frozen samples and electrical properties of water samples prepared by the two different methods, ‘contact’ and ‘non-contact’ were compared.

Based on these results, including results of the dielectric constant, redox potential measurement, polarity reversal, and cryogenic scanning electron microscopy, we propose a new three-dimensional heterogeneous water structure that includes high and low-density water zones.

## 2. Materials and methods

### 2.1. Preparation of structured-water samples

A hydrophilic silicate ceramic powder from the feldspar family, particle size ranging from 40 nm to 1 μm, was obtained from Quantum Energy Co., Ltd. Its average emissivity of far-infrared radiation is 0.927 for the range of 5 *μ*M ~ 20 *μ*M wavelengths and its emission power is 3.74 x 10^2^ W/m^2^∙*μ*M at 40 °C. To evaluate its hydrophilic nature, the zeta potential of the powder in water was measured by the electrophoretic light scattering method [22] (Photal Otsuka Electronics Zeta-potential and Particle size analyzer ELSZ-2).

Deionized water was obtained by a water purification system that produced ultra-pure water type I grade with an electrical resistivity of 18.2 MΩ∙cm and reduction of inorganics up to 99.99% (Young Lin Instrument, aqua MAX^™^ ultra 370 series). Structured water samples were prepared by two methods: contact treatment and non-contact treatment.

**Fig 1:**
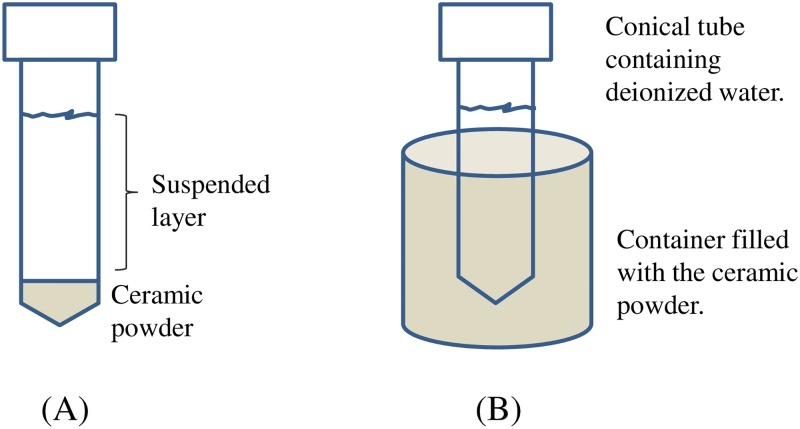
Schematic diagram showing the two sample preparatory methods. (A) Contact method: Deionized water mixed with the hydrophilic ceramic powder was agitated for 3 minutes and allowed to rest for 2 days. (B) Non-Contact method: A conical tube containing 50 mℓ of deionized water was inserted into the powder for 2 days.

In the contact treatment, the hydrophilic powder (1% in weight) was mixed with deionized water and agitated for 3 minutes with a magnetic stirrer. The solution was allowed to rest for two days, after which the water in the suspended layer (Fig 1A) labeled ‘contact water’ was used for various measurements except when it is specified.

In the non-contact treatment, a conical polyethylene tube containing 50 ml of deionized water was inserted into the powder for 48 hours, as shown in Fig 1B. For the dielectric constant measurements, the time was extended to 17 days. The water in the conical tube, so-exposed to the powder in a non-contact manner was used for various measurements.

### 2.2. Measurements of physical properties of water

Physical properties including density (Electronic densimeter MD-300S, ASTM D792), specific heat (Perkin-Elmer Pyris 1 DSC, KS M 3049), and surface tension (SEO Surface Tension Analyzer, Ring method) were measured both before and after the contact or non-contact treatments to investigate the change in these properties if any. Additionally, the dielectric constant of water was also measured by measuring the current between the outer and the inner cylinder of the probe (Nihon Rufuto dielectric constant meter 871). A low-distortion sine wave at a frequency of 10 kHz was applied to the outer cylinder of the probe.

Redox potential (oxidation/reduction potential) was measured (Dr DAQ redox sensor, TA137) by inserting the sensor into a 20 mℓ vial containing 10 mℓ of deionized water. The potential was recorded continuously during the non-contact treatment of more than 5 hours. The peak position of the redox potential was taken as a reference line for the evaluation of potential change. For example, the potential at 1 hour later from the peak position was adopted to calculate the decreased amount of potential and so on for 2, 3 and 5 hours.

Additionally, the redox potential of water samples mixed with the powder in three different concentrations, 1%, 3%, and 10%, was measured at 1 hour, 4 hours, and 8 hours after mixing. All measurements were carried out at room temperature, 23 °C.

### 2.3. Measurement of electric potentials and polarity

**Fig 2:**
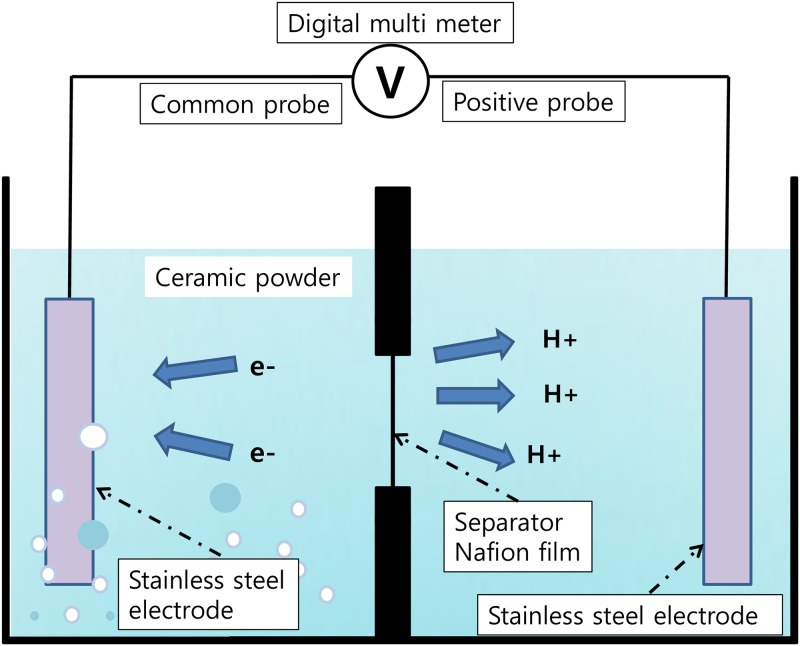
Schematic diagram of the potential measurement device. A polyethylene water container was divided into two sections by a wall in the middle. The holes were covered by the Nafion^®^ film or an aluminum foil separator. The two electrodes were placed at the extreme ends of the container.

A specially designed water container (23 cm x 16 cm x 9 cm) made of polyethylene with a wall in the middle was specially constructed for these measurements. The separating wall had two holes, each 5 cm in diameter. These were covered with Nafion^®^ membrane, NR-211, 25.4 μm thick (Ion Power, Inc.). Electrodes made of stainless steel were placed in each section respectively to measure the potential between the two sections. The electrodes were kept at the extreme ends of the container. The electric potentials between the two electrodes were measured with a multimeter (Hioki, 3803 Digital tester) and recorded in a computer through a data logger (Graphtec, midi logger GL220). Fig 2 shows the schematic diagram of this apparatus. The water container was filled with deionized water and left for one or two days until the electric potential was stabilized to near zero voltage. Thereafter, the hydrophilic ceramic powder was added to the left section. Measurements of electric potential between the two sections have been carried out at three different concentrations of 1%, 3%, and 10%, by pouring the corresponding amount of powder into the left section each time. The negative common probe of the multimeter was connected to the electrode placed in the left section of the container. We also investigated the effect of the membrane on the polarity of the potential by replacing the Nafion^®^ film with a 15 μm thick piece of aluminum foil as a separator in the container.

For the measurement of non-contact electric potential, 20 mℓ vials containing the required amount of the ceramic powder at each concentration were closed tightly and thoroughly rinsed on the outside with deionized water. These vials were placed in the left-hand section of the polyethylene container filled with deionized water. The electric potential between the two sections was then measured as usual (Fig 2).

### 2.4. Cryogenic field emission scanning electron microscopy of jet-frozen water samples

Water samples prepared in both ‘contact’ and the ‘non-contact’ manner (Fig 1A and 1B) were inserted into a sandwich-form copper carrier and quickly jet frozen by a jet freezing device (JFD 030, Bal-tec) in the presence of liquid nitrogen. The sample holder was fixed to a special device where freeze fracture, etching, and coating were performed in one unit (MED 020GBE, Bal-tec). Freeze-fracture was adopted for specimen preparation rather than cutting to observe fractured surface morphology. To increase the contrast of the fractured surface morphology, the fractured ice was etched at -110 °C for one minute and then, at the same temperature, coated with platinum for 100 seconds at 30 mA. A high vacuum cryogenic transfer system (VCT 100, Bal-tec) was used to insert the fractured sample into the field emission cryogenic scanning electron microscope (S-4700: Hitachi), maintained at -120 °C for low-temperature observations.

## 3. Results

### 3.1. Measurement of physical properties of water

The zeta potential of the ceramic powder was measured to be -37.7 mV, showing a good hydrophilic property. Other physical properties were evaluated and the results are summarized in Table 1. The water samples were treated as described in the remarks before measurement. Surface tension and density showed a slight variation within the scattering range without regarding the methods of treatment, i.e., ‘contact’ or ‘non-contact’ method. Specific heat showed a slight increase less than 1% after a non-contact treatment.

**Table 1:** Change of physical and electrical properties of water.

Sample preparation	Property	Before	After	Remarks
Mixed with the powder (contact method)	Surface tension	70.47 dyn/cm (SD 0.323)	70.37 dyn/cm (SD 0.161)	Water mixed with the powder (1%) was agitated for 2 hours. This was filtered with 2.5 μm grade 42 filter paper after 2 days.
	Density	996.00 kg/cm^3^ (SD 0.00)	1000.00 kg/cm^3^ (SD 0.00)	
	Dielectric constant	79.5 (SD 0.1)	113.7 (SD 4.2)	Samples were prepared in the same manner except for the powder concentration, 0.5% for this measurement. It was reduced due to the over limit of the conductivity of the mixed water sample.
Treated in a non-contact manner	Surface tension	71.60 dyn/cm (SD 0.000)	71.59 dyn/cm (SD 0.352)	48 hours of exposure time.
	Density	997.67 kg/m^3^ (SD 0.58)	996.00 kg/m^3^ (SD 0.00)	
	Specific heat*	4.255 J/g·°C	4.289 J/g·°C	
	Dielectric constant	81.1 (SD 0.1)	84.2 (SD 0.2)	10 days of exposure time.
			87.3 (SD 0.3)	17 days of exposure time.

* All of the data with SD (Standard Deviation) are the average of three times of measurement except the specific heat which was measured one time.

**Fig 3:**
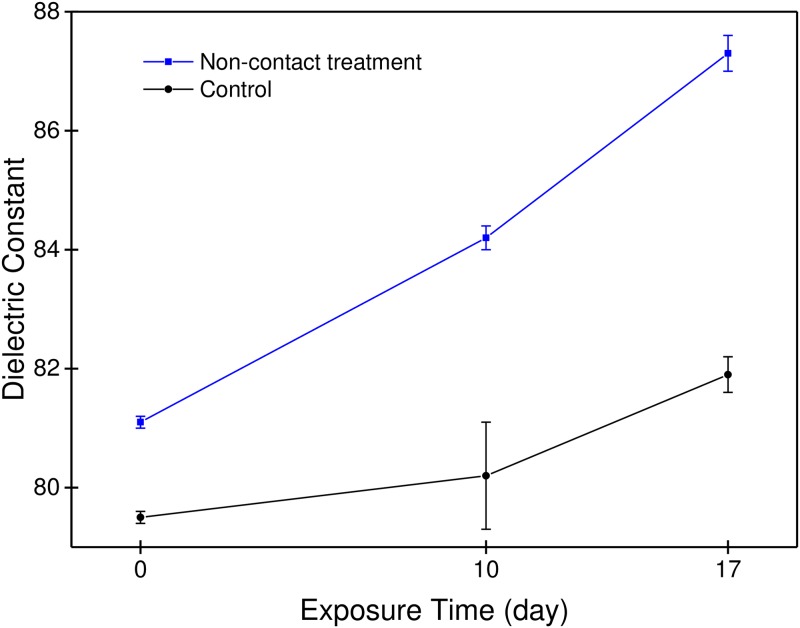
Change in dielectric constants with the length of exposure time. The water in a glass container was treated in a non-contact manner as described in Fig 1B. The dielectric constant of the control water has increased by 3.0% while that of the non-contact treated water has increased by 7.6% for the same time duration, 17 days.

The dielectric constant of pure water was measured to be 79.5 (measured at a frequency of 10 kHz) and this value increased to 113.7 after mixing 0.5% of the powder. For non-contact treated water, the dielectric constant increased from 81.1 up to 87.3 with the length of the exposure time, 17 days, to the powder. This amount of change corresponds to 7.6% increase. While that of the control water, no treatment, increased to 81.9 from 79.5 for the same time duration as shown in Fig 3, which corresponds to 3.0% increase.

**Fig 4:**
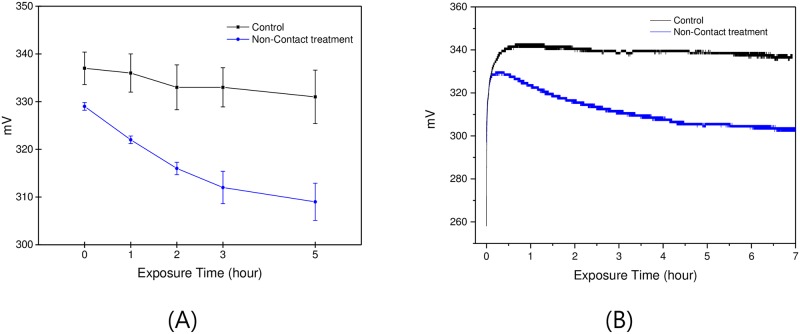
Change in redox potential with the length of exposure time. (A) Redox potential decreased continuously from 329 mV to 309 mV during the 5 hours of additional exposure time after it reached the peak position. For the control water, it decreased from 337 mV to 331 mV for the same time duration. The value at the 0 hour of exposure time indicates the potential at the peak position. (B) Typical patterns of change in redox potential of the control and the non-contact treated waters. The potential decreases from the beginning of non-contact exposure.

The redox potential decreased for the water treated in a non-contact manner as shown in Fig 4A, which corresponds to 6.1% reduction, after additional 5 hours of exposure time from the peak position. In contrast, the control group showed a 1.8% reduction for the same time duration. It took usually 20 to 30 minutes to reach the peak position and then it began to decrease as shown in Fig 4B. In overall, the potential, as well as the time at the peak position, were smaller (329 mV compared to 337 mV) and shorter (933 s compared to 1486 s) in average for the non-contact treated water compared to the control.

The water samples mixed with the powder showed the same trend, decreasing of redox potential with the time. For example, the potential of water mixed with 1% of powder decreased to 285 mV, 262 mV, and 266 mV after 1 hour, 4 hours, and 8 hours later, respectively. The water showed 312 mV of redox potential before mixing. Interestingly, there was almost no difference in the potential among the three water samples having different concentrations, 1%, 3%, and 10%. The difference among them was at most 3 mV.

### 3.2. Electric potential and polarity measurement

**Fig 5:**
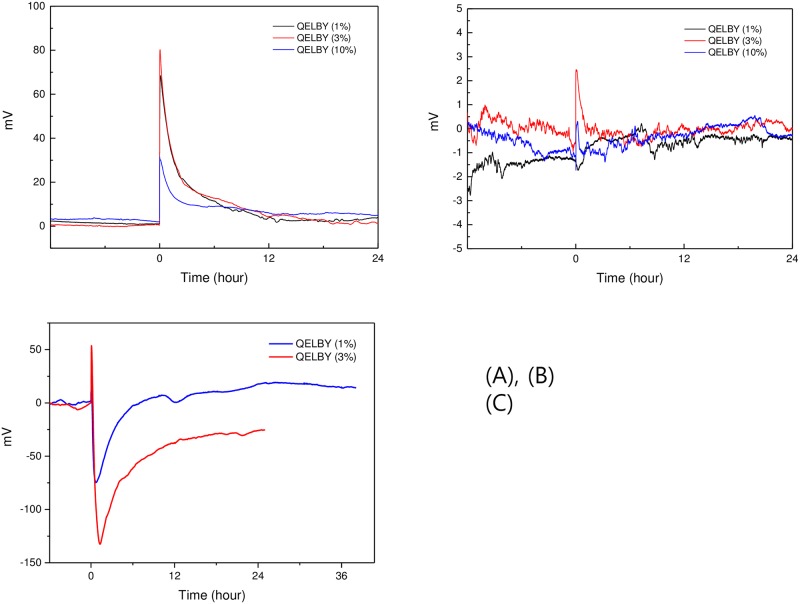
Electric potential measurement of water. (A) Nafion^®^ film was used as a separator between the two sections. The corresponding amount of powder was mixed with the water at the 0 hour time-point. (B) Potential generation in a non-contact manner. Nafion^®^ film was used as a separator between the two sections. The vials were inserted into the water at the 0 hour time-point. (C) Aluminum foil was used as a separator between the two sections. The powder was mixed with the water at the 0 hour time-point. Note that the peak polarity has been reversed compared to the results shown in (A) and (B).

One of the most important characteristics of the exclusion zone (EZ) is the electric potential difference across the boundary between the EZ and the bulk water outside of this zone. When water was mixed with the hydrophilic ceramic powder, we expected the negative OH^-^ components/charges of the EZ and protons to be similarly separated at the interface of the particles between the EZ water and bulk water. Fig 5A shows the results of the potential measurement for 1%, 3% and 10% concentration by weight of the powder in deionized water, respectively. Since the Nafion^®^ film is a good proton conductor, the protons will pass through the Nafion^®^ film from left to right section and a positive potential difference will be measured between the two electrodes (Fig 2). Results were as expected. When 1% of the powder by weight was mixed with 500 mℓ of deionized water in the left section, a peak climaxing to +68 mV at the positive probe was observed right after mixing (Fig 5A). For a 3% powder concentration, the peak climaxed at +80 mV. On the other hand, the height of the peak at 10% powder concentration, +30 mV, was considerably lower than the other two peaks.

We also confirmed the development of an electric potential in a non-contact manner. This was done by immersing the closed vials containing the ceramic powder into the left section of the container. As shown in Fig 5B, the vial containing 1% of the ceramic powder did not show any increase in potential when it was immersed in the left section. At 3%, a clear peak was observed right after immersion. Interestingly, the peak height generated by the vials containing 10% powder was again lower than those containing 3% of the powder. These results were similar to those obtained with the contact method. Furthermore, all concentrations displayed a similar tendency to decrease in potential with time.

When aluminum foil was used as a separator between the two sections of the container, the electric polarity was reversed as shown in Fig 5C. At the beginning right after the pouring of the powder, an increase in the positive direction was observed and reached the maximum level +25 mV and +53 mV at 1% and 3% concentration of the powder, respectively. However, it declined sharply and reached the minimum after 0.5 hours and 1 hour at 1% and 3% concentration, respectively, to the level -75 mV and -130 mV.

### 3.3. Cryogenic scanning electron microscopy

**Fig 6:**
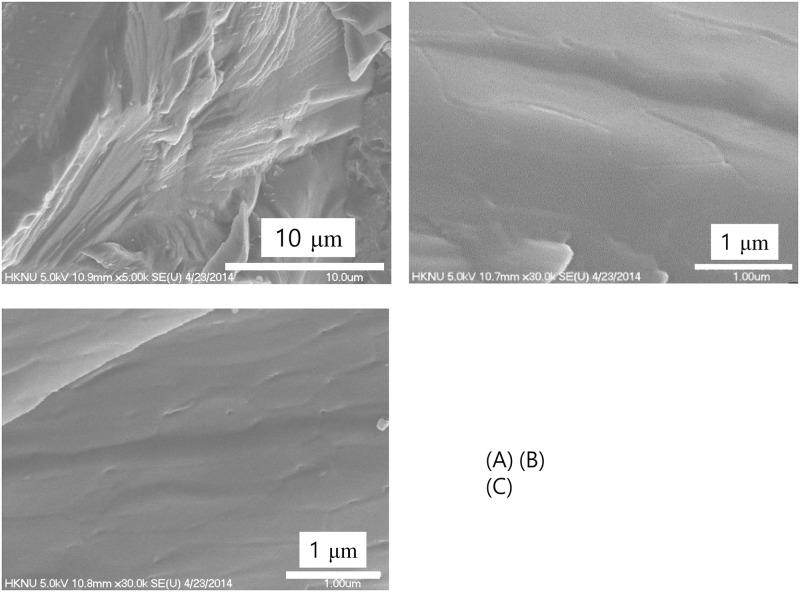
Deionized water. Fractured surface of the ice as observed by cryogenic scanning electron microscopy produced by jet-freezing and in-situ breaking.

**Fig 7:**
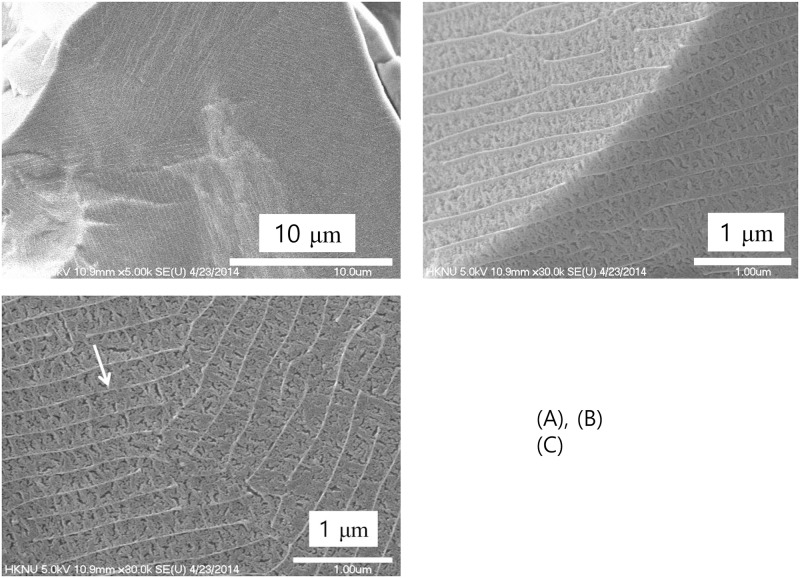
Contact water. **Fractured surface of the ice as observed by cryogenic scanning electron microscopy produced by jet-freezing and in-situ breaking**. The arrow in (C) indicates the bright boundary.

**Fig 8:**
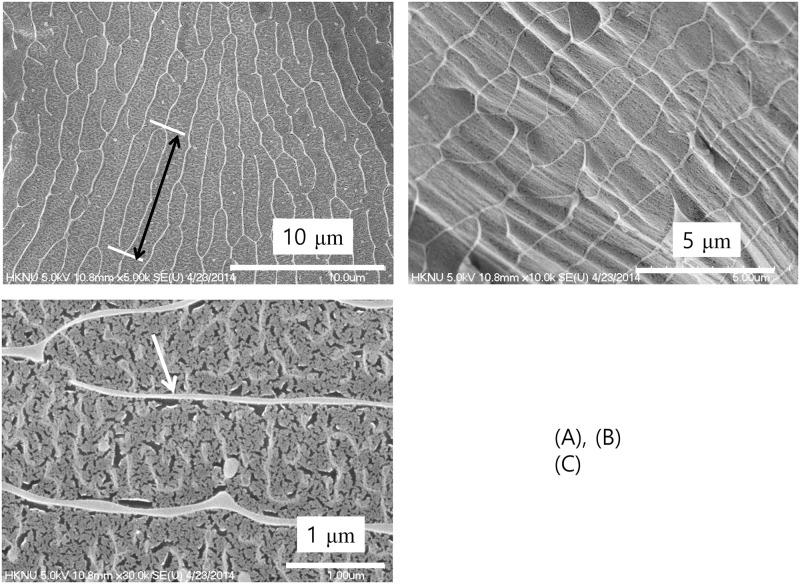
Non-contact water. **Fractured surface of the ice as observed by cryogenic scanning electron microscopy produced by jet-freezing and in-situ breaking**. Black arrow in (A) indicates the longitudinal length of a unit cell. This low magnification shows the overall pattern of the unit cell. The arrow in (C) indicates the bright boundary.

**Fig 9:**
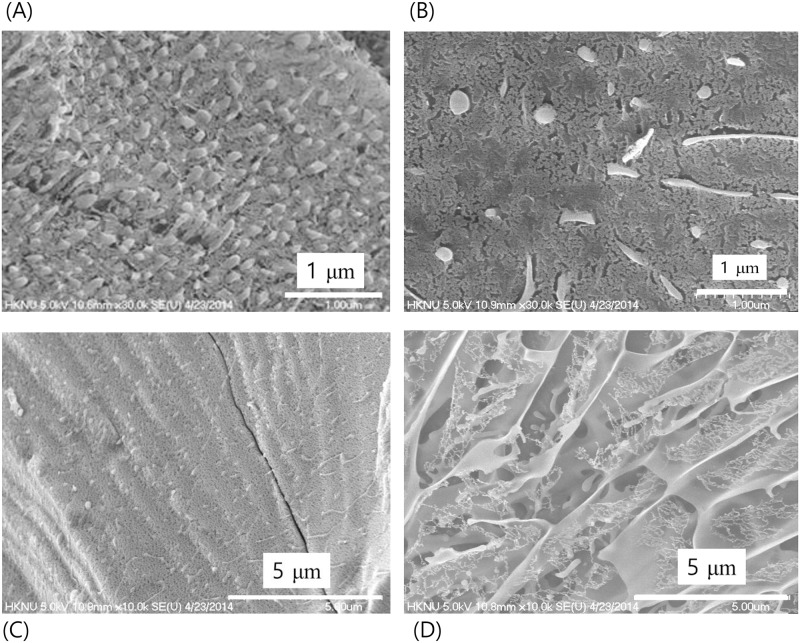
String and wall-like form of ice from water treated in a non-contact manner. These images were obtained from different locations of the same sample. (A) String form of ice that appeared as fibers. The diameter of the fiber was estimated to be 0.1 μm ~ 0.2 μm. (B) Co-existence of string form and cell-like boundary. (C) A possible transition stage of string form to cell-like form as the ‘degree of structure’ increases. (D) The three-dimensional cell-like structure resembling a sponge.

Figs 6–9 show results of cryogenic scanning-electron microscopy of *in-situ* fractured and etched ice surface of water samples. Deionized water was used as a control and it revealed a rough and flat fractured surface (Fig 6A) with weak traces of boundaries in ~nm range and no notable special morphology (Fig 6B and 6C).

On the other hand, ice formed from the water in contact with the powder (Contact method; Fig 1A), displayed clear, bright boundaries, as indicated by the white arrow in Fig 7C. The brightness indicated an area where hardness is greater than other areas because it was not etched as much. Possibly, these boundaries correspond to EZ water, whose density exceeds that of bulk water. The thickness of the bright boundary measured to be less than 0.01 μm. On an average, the width between the bright boundaries was about 0.3 μm and the length of the unit cell exceeded 10 μm.

Fig 8 shows the fractured surface of the ice frozen from deionized water treated in a non-contact manner (Non-contact method; Fig 1B). Surprisingly, while the basic shape was similar to that observed in Fig 7, the boundary was thicker, in the range of 0.03 μm ~ 0.15 μm, as indicated by the white arrow in Fig 8C. Additionally, the unit cell size was larger in width and length for the ice obtained from the water treated in a non-contact manner. The unit cell was about 1 μm in width and approximately 15 μm in length with an oval shape (black arrow in Fig 8A). The largely similar appearance indicated that even though the two waters were prepared using different methods, they possessed the same structural pattern.

Detailed investigations on the fracture surface of the ice from water treated in a non-contact manner further revealed that exclusion zone water could exist in the form of strings or fiber-like structures (Fig 9A) depending on the area of observation. The string may develop in its own structure as shown in Fig 9A and sometimes they are interconnected with each other to form a cell-like structure. Co-existence of these forms can be observed as shown in Fig 9B and 9C. The well-developed three-dimensional cell-like structure resembles a sponge as depicted in Fig 9D.

## 4. Discussion

### 4.1. Heterogeneous water structure

In this study, the hydrophilic ceramic powder was used to prepare water samples using two different methods—contact and non-contact. With the contact method, we obtained a normal-sized exclusion zone (~100 μm) around the ceramic particles suspended in water, similar to that reported by Sharma et al [13]. In cryogenic scanning electron microscopy studies of frozen samples of water prepared by the two methods, we observed clear, bright boundaries that enclosed low-density ordinary water to form cell-like structures. These boundaries designate regions of high-density water in EZ that survived mechanical impacts including etching in procedural handling. We also found that the morphology of the ice structure was similar regardless of the method of sample preparation, contact or non-contact. The only difference being the scale of the unit cell size and thickness of the cell boundary. Taken as an index of the “degree of structure,” the unit cell size was reduced in ice obtained when the water had been mixed with the powder. We speculate that direct contact of the powder with water resulted in a large number of nucleation sites for the formation of dense structured water, i.e., a higher degree of structure [23].

Recent studies conducted with X-ray and neutron scattering have reported the coexistence of high- and low-density water at ambient temperatures [3–8]. It has been further proposed that high- and low-density water coexist in a metastable phase [5]. Additionally, the thickness of the high-density water has been reported to be in a range of ~nm scale [3–9]. Compare to these reports, the thickness of the boundary in Fig 8C shows that it can be up to a hundred nm scale. Fig 6B and 6C provide a clue to understanding this difference. The ice obtained from the control water shows a very weak trace of boundary morphology which is about several nm scale thickness. Light and infrared radiations are found to increase the size of the exclusion zone formed in water [24]. Probably, the thickness of the high-density water has been increased, especially in a non-contact treatment, by the far-infrared waves from the hydrophilic ceramic powder. The thin layer of the high-density water in control water played as a foundation for the growth of the thick high-density water boundary with the help of the far-infrared waves. If water has a well-developed heterogeneous structure is used for the study of water structure, the results would be different.

Fig 9A shows that the exclusion zone can be in a form of strings or fiber-like structure. From the photograph shown in Fig 9B and 9C, where the parts of the boundary of cell-like structure and string-like structure coexist, it seems that they are interconnected and form the cell-like network eventually. This observation in this study is quite interesting since it was claimed that water molecules exist as small hydrogen-bonded strings and three-dimensional hydrogen-bonded network by neutron diffraction [25]. Although it is about alcohol-water mixture, it shows the possibility that the high-density water in exclusion zone may be present in string form.

The formation of structured water in a non-contact manner in the present study also implies a role for infrared radiation from the ceramic powder. The well-known biological effects of the far-infrared wave may be interpreted from the point of view of assisting the formation of the structured water, EZ, in biological terrain. Undoubtedly, further research will be required to deal with this issue considering the complexity of the water structure and its biological effects [19–21, 26–28].

### 4.2. Electrical properties of heterogeneous structured water

As the water is structured, we find distinct changes in the physical properties of water (Table 1), especially the dielectric constant and redox potential. In the non-contact setting, a lengthier exposure to the powder increased the dielectric constant and decreased redox potential, which indicates the continued formation of structured water with the passage of time. The increased dielectric constant is related to the increased charge storage capacity of water due to the increased interfacial area between the EZ and ordinary water outside of it as it is structured.

Contrary to the dielectric constant, the redox potential reached the saturation level after 4 ~ 5 hours of exposure time. Decreased redox potential means that the water may show an antioxidant property when it is mixed with oxidant materials. Actually, it was confirmed by the ultra-weak photon emission measurement that the non-contact treated water shows lower photon emission compared to control when it is mixed with a strong oxidant, 10 mM tert-Butyl hydroperoxide (TBHP) [21]. In this case, the difference between the control and the non-contact treated water became larger after 4 days of treatment.

The water mixed with the powder also showed decreased redox potential. However, there was almost no difference among the three water samples with different concentrations of powder, 1%, 3% and 10%. It seems that far lower concentration of powder, maybe in the ppm range, is enough to observe the same phenomenon [21].

Unlike the electrical properties, it was found that those physical properties such as surface tension, density, and specific heat are not so sensitive to the change in water structure. These results imply that depending on the sensitivity of measurement methods, the ranges of detection limits are different even though the water is structured gradually for longer times. For example, redox potential did not change after several hours of exposure, however, dielectric constant and ultra-weak photon emission showed a continuous change even after several days of non-contact treatment.

Earlier studies [29] reported the development of a negative electric potential as high as -150 mV to -200 mV across the boundary between exclusion zone and bulk water. We obtained comparable results when electrodes were inserted into the two sections of the water container separated by a Nafion^®^ film, implying a similar mechanism. A sharp and immediate positive increase in the electric potential was observed when the powder was poured into the left section of the container containing the electrode connected to the common pole of the multimeter. This phenomenon implies an immediate formation of structured water with abundant protons outside the exclusion zone. Being a good proton conductor, the Nafion^®^ film could pass those protons through to the right section of the container and result in an increased electric potential in the positive direction at the electrode in the right section.

Earlier studies implied that protons could penetrate through exclusion-zone gaps on both sides of the Nafion^®^ film [30]. Possibly, the driving force arising from the difference in proton concentration between the two sections could be larger than the exclusive force of the exclusion zone. However, more detailed experimentation will be needed to identify how proton penetration is occurring in these gaps. Additionally, it seems that there is an optimum concentration of powder for the formation of structured water and the generation of the electric potential from the fact that the electric potential has been decreased for 10% concentration of powder. Definitely, it deserves further research work.

On the other hand, for the container with the aluminum foil separator, the polarity was reversed; it reached -75 mV and -130 mV at 1% and 3% concentration of the powder, respectively. The negative charge generated in the exclusion zone apparently moved to the right section of the container through the conductive aluminum foil and results in the reversed polarity. The increased availability of electrons by the formation of structured water can be confirmed by the decreased redox potential and the increased antioxidant property of water [21].

### 4.3. A new model of heterogeneous water structure

**Fig 10:**
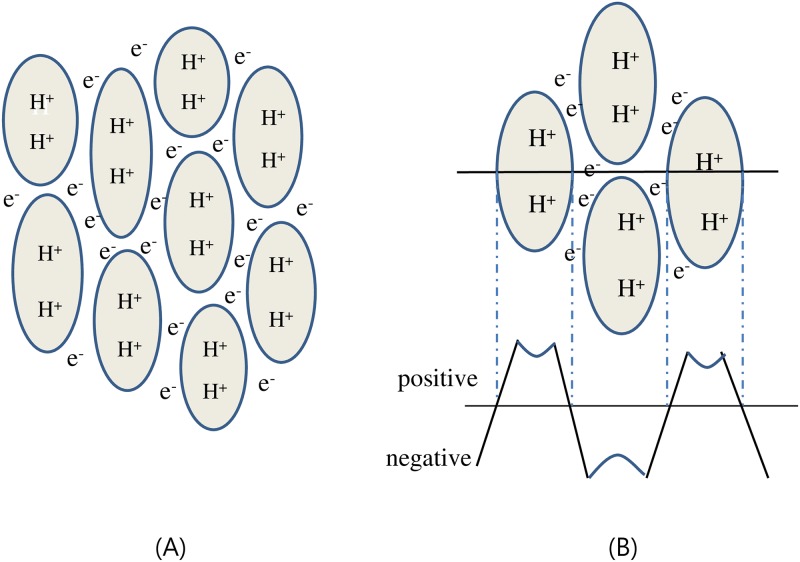
Schematic diagram of the heterogeneous structure of water. (A) A cell-like arrangement of water, with high-density water (exclusion zone) forming the walls (indicated with e^-^) and low-density ordinary water residing within the walled boundaries (indicated with H^+^). (B) Electric potential distribution across the boundary. High-density water in exclusion zone has negative electric potential while ordinary low-density water has positive polarity.

In sum, based on the development of electric potential across the interface between the exclusion zone and bulk water, as well as the three-dimensional networked cell-like structures observed in the presence of the powder, we propose a new heterogeneous model for a bulk water (Fig 10A), which allows us to understand the relationship between its structure and some physical properties. Here, the exclusion-zone, composed of high-density water, forms the wall of the cell-like structure, while the low-density ordinary water resides within each cell. The exclusion-zone has a negative charge, while the ordinary water inside the cell has a positive electric potential.

Fig 10B shows the expected change in electric potential across the boundary between the exclusion zone and ordinary water if the potential is measured along the horizontal line. As the size of a unit cell becomes smaller, the ‘degree of structure’ as well as the sum of all interfacial areas between the high-density water and the low-density water increases. From this model, the increased dielectric constant of water treated in a non-contact manner can be understood in terms of the ‘degree of structure’, which increases with exposure time. Increased ‘degree of structure’ means the smaller cell size and a hence larger total interfacial area between the exclusion zone and ordinary water which results in the increased charge storage capacity and antioxidant property.

## 5. Conclusion

Water is reported to have a heterogeneous structure composed of high- and low-density water. Using cryogenic scanning electron microscopy, we have demonstrated the presence of a cell-like structure where the high-density water exists like a wall. Low-density ordinary water resides in between. From the measurement of electric charge separation, it is strongly indicated that the origin of the high-density water is the exclusion zone. The proposed model can suitably explain the anomalous properties, especially the electrical property of water. With decreasing unit cell size, the ‘degree of structure’ increases resulting in an increased charge capacity of the structured water due to the augmented total interfacial area. At the same time, due to the separation of electrons, the redox potential and the antioxidant property have been increased. Our conclusions are well supported by measurements of electric potential that occur due to charge separation at the interface along with the formation of an exclusion zone of water. On the other hand, it was found that those physical properties such as surface tension, density, and specific heat are not so sensitive to the change in water structure.
